# The Amyloid State of Mutant p53 in Cancer

**DOI:** 10.31662/jmaj.2026-0134

**Published:** 2026-07-24

**Authors:** Naoyuki Iwahashi, Kazuhiko Ino

**Affiliations:** 1 Department of Obstetrics and Gynecology, Wakayama Medical University, Wakayama, Japan

**Keywords:** p53, protein aggregate, oncology, cancer, gene mutation

## Abstract

Mutations in *TP53*, the most frequently altered gene in human cancer, have long been interpreted within a genetic framework centered on loss of tumor-suppressive function and dominant-negative inhibition. Here, we highlight an emerging paradigm in which mutant p53 operates as a protein conformational disease. A growing body of evidence demonstrates that many cancer-associated p53 mutants adopt an amyloid-like state driven by structural destabilization of the DNA-binding domain, exposing aggregation-prone sequences that nucleate β-sheet–rich oligomers and fibrils.

These aggregates exhibit prion-like properties, enabling templated misfolding and self-propagation of aberrant p53 conformations within cells. Beyond intracellular accumulation, recent experimental findings suggest that p53 aggregates may be transferred between cells via extracellular vesicles and heparan sulfate proteoglycan–mediated uptake. However, whether such transfer results in sustained tissue-level propagation within human tumors remains to be established. This proposed “seed-and-spread” model provides an emerging conceptual framework for understanding tumor heterogeneity and disease evolution beyond genetic alterations alone.

Clinically, amyloid-like p53 aggregates have been detected in multiple malignancies, including ovarian, breast, and lung cancers, where they are associated with aggressive phenotypes, therapeutic resistance, and poor patient outcomes. These observations indicate that the conformational state of p53 represents a critical but previously underappreciated determinant of tumor behavior.

Reframing mutant p53 as a self-propagating oncogenic proteinopathy bridges cancer biology with the principles of neurodegenerative disease and opens new therapeutic opportunities. Targeting the aggregation process, restoring native folding, promoting degradation of misfolded p53, or interrupting intercellular transmission may enable direct intervention against *TP53*-mutant cancers, which account for nearly half of all human malignancies. This perspective establishes protein misfolding and conformational propagation as central drivers of oncogenesis and highlights a new frontier for precision oncology.

## Introduction

The tumor suppressor p53 is a key regulator of genomic stability, coordinating cellular responses to stresses such as DNA damage, oncogene activation, and metabolic perturbation ^(1), (2)^. Acting as a transcription factor, p53 controls pathways involved in cell-cycle arrest, apoptosis, senescence, and DNA repair, thereby serving as a major barrier to malignant transformation. Consistent with this essential role, *TP53* is the most frequently mutated gene in human cancer, with alterations present in approximately half of all malignancies ^(3)^. Traditionally, the oncogenic consequences of* TP53* mutations have been explained by loss of tumor-suppressive function and dominant-negative inhibition of wild-type p53 ^(4), (5)^. Increasing evidence indicates that mutant p53 can acquire gain-of-function activities that actively promote tumor progression, including transcriptional rewiring, chromatin remodeling, and enhanced invasion and metastasis ^(6), (7), (8)^. However, the molecular mechanisms underlying these oncogenic phenotypes remain incompletely understood.

A growing body of evidence has introduced a new perspective on mutant p53 biology, revealing that many cancer-associated p53 mutants possess an intrinsic propensity to misfold and assemble into amyloid-like protein aggregates ^(9), (10), (11)^. Destabilization of the p53 DNA-binding domain exposes aggregation-prone regions that promote the formation of oligomers and β-sheet–rich fibrils. Importantly, these aggregates can exhibit prion-like properties, acting as conformational templates that induce further p53 aggregation. Evidence further suggests that extracellular p53 aggregates can interact with heparan sulfate proteoglycans (HSPGs) on the cell surface, facilitating their uptake and transmission between cells ^(12)^. Such mechanisms may allow aggregation-prone p53 species to disseminate within tumor tissues and influence the tumor microenvironment.

In this review, we discuss the emerging concept that selected mutant p53 proteins can adopt amyloid-like aggregated states with prion-like biochemical properties and evaluate how these processes may contribute to cancer progression (Figure 1). We examine the structural basis of p53 aggregation, the experimental evidence and limitations regarding its propagation, and therapeutic strategies aimed at targeting this previously underappreciated aspect of mutant p53 biology. To avoid overinterpretation, we use the term “prion-like” to refer to shared biochemical properties, such as templated misfolding, seeding activity, and aggregate uptake, rather than to imply infectivity, strain behavior, or fulfillment of all criteria of classical prions. We also distinguish related terms throughout the review: “amyloid-like p53 assemblies” refers to species with amyloid-related biophysical properties, “aggregated p53” broadly denotes misfolded p53 assemblies, and “cytoplasmic p53 aggregates” refers to aggregated p53 detected within the cytoplasm of tumor cells.

**Figure 1.**
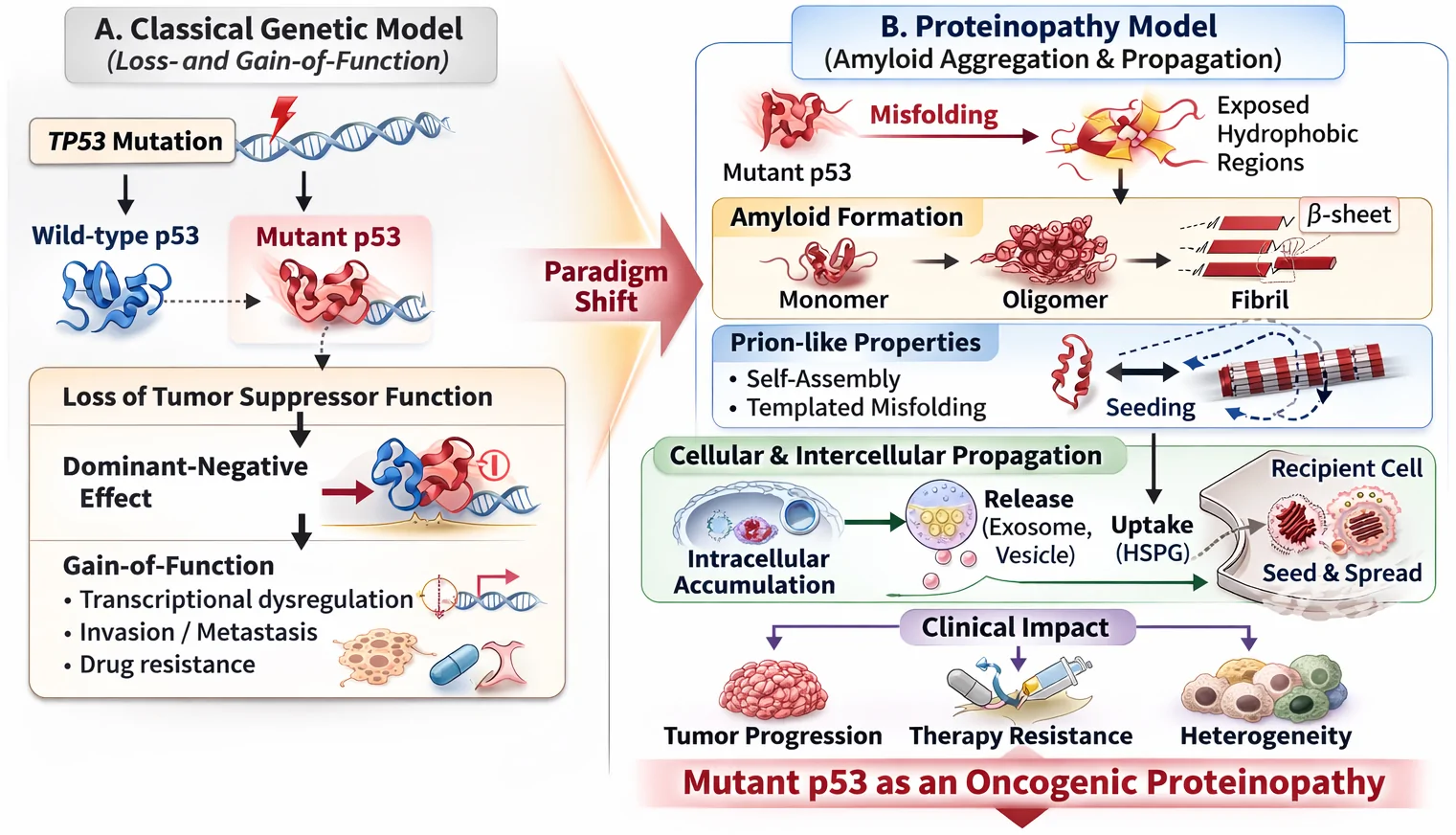
Paradigm shift in mutant p53 biology from a genetic model to a conformational proteinopathy. The left panel illustrates the classical genetic model of mutant p53, in which *TP53* mutations lead to loss of tumor suppressor function, dominant-negative effects, and gain-of-function activities that promote tumor progression. The right panel shows the emerging proteinopathy model, in which mutant p53 undergoes misfolding and forms amyloid-like oligomers and β-sheet–rich fibrils with prion-like properties, enabling self-assembly and templated aggregation. These aggregates can accumulate intracellularly and propagate between cells via extracellular vesicles and heparan sulfate proteoglycan–mediated uptake, consistent with a “seed-and-spread” mechanism. This conceptual shift highlights mutant p53 as a self-propagating oncogenic proteinopathy that contributes to tumor progression and therapeutic resistance.

## Structural Basis of the Amyloid State of p53

The structural integrity of p53 is essential for its tumor suppressor function. p53 acts as a tetrameric transcription factor composed of several domains, including the N-terminal transactivation domain, the central DNA-binding domain, the oligomerization domain, and a regulatory C-terminal region. Among these, the DNA-binding domain forms the structural core of the protein and harbors the majority of cancer-associated mutations ^(5)^. The p53 DNA-binding domain is intrinsically marginally stable, a property that allows conformational flexibility required for DNA binding but also makes the protein susceptible to destabilization. Structural studies indicate that many cancer-associated missense mutations cluster within the p53 DNA-binding domain, particularly at hotspot residues such as R175, R248, and R273, where they disrupt critical electrostatic interactions and hydrophobic packing ^(13), (14), (15)^. This destabilization reduces the thermodynamic stability of the domain and increases its propensity to adopt partially unfolded conformations.

Destabilization of the p53 DNA-binding domain exposes normally buried hydrophobic surfaces, promoting protein self-association ^(14), (15)^. Biophysical studies have shown that mutant p53 can assemble into soluble oligomeric intermediates that subsequently convert into ordered fibrillar aggregates with classical amyloid features, including β-sheet enrichment, binding to amyloid-specific dyes such as thioflavin T, and resistance to proteolytic degradation. These aggregates are thought to arise from aggregation-prone segments within the DNA-binding domain that become exposed upon destabilizing mutations, initiating nucleation and fibril growth through partially unfolded intermediates. The aggregation process likely proceeds through multiple states, beginning with misfolded monomers, followed by oligomer formation, and ultimately leading to amyloid fibrils that can accumulate into larger inclusion bodies within tumor cells. Recent studies further suggest that liquid–liquid phase separation may facilitate this process by concentrating mutant p53 within dynamic condensates that promote nucleation of ordered aggregates, which can subsequently mature into stable amyloid assemblies ^(16), (17)^. Importantly, mutant p53 aggregates can also sequester other members of the p53 family, including p63 and p73, whose homologous DNA-binding domains enable their incorporation into mixed aggregates, thereby suppressing their transcriptional activity and contributing to the dominant gain-of-function phenotypes observed in many *TP53*-mutant cancers ^(7), (9), (18)^.

Collectively, these structural insights indicate that selected cancer-associated *TP53* mutations can shift p53 from a functional transcription factor to an aggregation-prone protein capable of forming amyloid-like structures. Such assemblies may impair normal p53 activity and create seeds for further aggregation, providing a mechanistic foundation for the prion-like biochemical behavior discussed in subsequent sections. It should also be emphasized that not all *TP53* mutations are expected to promote p53 aggregation. Aggregation-prone behavior has been most closely linked to missense mutations that destabilize the DNA-binding domain while allowing production of a full-length or partially stable mutant protein ^(9), (10), (11), (14), (15)^. In contrast, DNA-contact mutations, such as R248Q/W and R273H/C, primarily impair DNA binding and may show more context-dependent aggregation propensity ^(13), (14), (15)^. Nonsense mutations, frameshift mutations, deletions, or alterations that markedly reduce p53 protein expression may inactivate p53 through loss-of-function mechanisms without generating aggregation-prone protein species ^(5)^. Thus, the aggregation-based model does not replace classical mechanisms of p53 inactivation but rather adds an additional conformational layer that may apply to selected subsets of *TP53*-mutant cancers (Table 1).

**Table 1.** Representative TP53 Mutation Classes and Their Relevance to p53 Aggregation.

Mutation class	*TP53* mutation status	Main molecular consequence	Expected relevance to p53 aggregation	Evidence status
Structurally destabilizing missense mutants	R175H, G245S, R249S, R282W	Destabilize the DNA-binding domain while usually allowing production of full-length mutant p53.	Relatively high. Destabilization can expose aggregation-prone sequences and promote amyloid-like oligomers or fibrils.	Supported mainly by biochemical and cellular evidence. Clinical relevance is mutation- and tumor-context dependent.
DNA-contact missense mutants	R248Q/W, R273H/C	Primarily impair DNA binding; structural destabilization is variable and may be less prominent than in structural mutants.	Context dependent. Some contact mutants may aggregate under selected conditions, but this should not be assumed universally.	Evidence is more variable. These mutants should be distinguished from structurally destabilizing mutants.
Oligomerization or localization-altering variants	Tetramerization domain or nuclear localization signal region alterations	May affect tetramerization, nuclear localization, or intracellular distribution of p53.	Mechanism may differ from classical full-length DNA-binding domain-driven amyloid-like aggregation.	Limited and emerging evidence. Relevance should be interpreted according to the specific alteration and retained protein product.
Truncating or loss-of-expression alterations	Nonsense mutations, frameshift mutations, large deletions	Often cause premature termination, reduced protein expression, truncated protein, or p53-null phenotypes.	Generally low for classical full-length p53 aggregation, unless a stable aggregation-prone truncated protein is produced.	Usually reflects loss-of-function mechanisms distinct from amyloid-like aggregation.
Wild-type under specific stress contexts	Wild-type	Normally functions as a regulated transcription factor; misfolding may occur under selected experimental or stress conditions.	Not generalizable as a cancer-wide mechanism.	Experimental or limited evidence. Should not be equated with aggregation-prone mutant p53 in tumors.

## Prion-Like Propagation of Mutant p53

Before discussing the prion-like behavior of mutant p53, it is important to clarify the evidentiary framework used in this review. We distinguish three levels of evidence. First, broadly established findings include the high frequency of *TP53* mutations in human cancers, the loss of tumor-suppressive function, dominant-negative effects, and gain-of-function phenotypes of selected mutant p53 proteins. Second, supported but context-dependent observations include the ability of several cancer-associated mutant p53 proteins, particularly structurally destabilized missense mutants, to form amyloid-like assemblies in biochemical and cellular systems. Third, speculative or early-stage models include the possibility that p53 aggregates propagate through tumor tissues in vivo and contribute to spatial tumor evolution in a manner analogous to, but distinct from, neurodegenerative proteinopathies. Throughout this review, we have revised the text to reflect these distinctions and to avoid assigning equivalent evidentiary weight to these categories.

The ability of mutant p53 to form amyloid-like aggregates has raised the intriguing possibility that p53 may propagate through mechanisms reminiscent of prion biology ^(9), (10), (12)^. Prions are misfolded proteins that convert normally folded molecules into pathogenic conformations through templated structural conversion, thereby amplifying protein aggregation in a self-perpetuating manner. Accumulating experimental evidence suggests that mutant p53 aggregates share several features of this prion-like behavior. In particular, preformed aggregates can accelerate the aggregation of soluble p53 through nucleation-dependent mechanisms in which aggregated species act as structural templates that promote the conversion of native or partially folded p53 into aggregation-prone conformations. Cellular experiments support this model, demonstrating that introduction of aggregated p53 into cultured cells can induce aggregation of endogenous p53 and drive the formation of intracellular inclusions even in the absence of additional genetic alterations. Once nucleated, aggregation proceeds through progressive recruitment of soluble p53 molecules, ultimately generating large cytoplasmic assemblies enriched in β-sheet–containing fibrillar structures characteristic of amyloid aggregates.

The propagation of mutant p53 aggregates may have important implications for tumor biology. Aggregated p53 species can persist as stable seeds that continuously recruit soluble p53, thereby promoting the gradual accumulation of aggregation-prone conformations within tumor cells ^(9), (10)^. In addition to direct uptake of extracellular aggregates, emerging evidence suggests that intercellular transmission of aggregation-prone proteins may also occur through extracellular vesicles, including exosomes, which can transport misfolded protein assemblies between cells ^(19), (20), (21)^. Such vesicle-mediated transfer has been described for several amyloidogenic proteins and may similarly contribute to the dissemination of mutant p53 aggregates within tumor tissues. Mutant p53 aggregates may also incorporate other members of the p53 protein family, including p63 and p73, further reinforcing their dominant oncogenic effects. Conceptually, this behavior parallels propagation mechanisms described in several neurodegenerative proteinopathies ^(22), (23), (24)^, in which proteins such as tau, α-synuclein, and amyloid-β spread through templated misfolding and the spatial transmission of aggregated conformations across tissues. These similarities suggest that common biophysical principles may govern the propagation of misfolded proteins across distinct disease contexts and support a model in which mutant p53 aggregates behave as self-propagating assemblies that accumulate within tumor cells and contribute to tumor progression.

It should be emphasized that the current evidence for prion-like behavior of p53 is strongest at the biochemical and cellular levels. Preformed p53 aggregates can seed further aggregation of soluble p53, and extracellular aggregates can be internalized by recipient cells in experimental systems. However, unlike neurodegenerative proteinopathies, long-range tissue-level propagation of p53 aggregates within human tumors remains unestablished. Thus, the term “prion-like” is used here in an operational sense to describe selected biochemical and cellular behaviors, including seeding, templated misfolding, and aggregate uptake, rather than to imply infectivity, strain behavior, or fully established spread at the organismal or tissue level.

## Heparan Sulfate–Mediated Transmission of p53 Aggregates

Propagation of aggregation-prone proteins often depends on mechanisms that enable their transfer between cells. In several protein misfolding disorders, pathogenic protein assemblies can be released from donor cells and subsequently interact with recipient cells through specific uptake pathways ^(20), (23)^. One important mechanism involves HSPGs, which are abundant components of the plasma membrane and extracellular matrix ^(25)^. HSPGs consist of core proteins decorated with sulfated glycosaminoglycan chains capable of binding a wide range of ligands, including misfolded protein assemblies. Extracellular aggregates frequently interact with these negatively charged heparan sulfate chains, facilitating their retention on the cell surface and promoting endocytic uptake. Emerging evidence suggests that mutant p53 aggregates exploit similar pathways ^(12), (26)^. Aggregated p53 species can bind sulfated glycosaminoglycans through electrostatic interactions between positively charged regions of the protein and negatively charged sulfate groups on heparan sulfate chains, thereby enhancing their association with the cell surface and internalization through endocytic pathways. The tumor microenvironment may further facilitate this process ^(27), (28), (29)^. Cancer tissues frequently exhibit extensive extracellular matrix remodeling and increased expression of cell-surface proteoglycans, including syndecans and glypicans, which enhance the capture and internalization of protein aggregates. Such changes can create a permissive environment that favors the retention, uptake, and intracellular seeding of aggregation-prone proteins. Together, these observations support a proposed seed-and-spread model in which aggregated p53 may function as a pathogenic seed, while the tumor microenvironment may provide a permissive environment for its retention, uptake, and local dissemination. However, direct evidence that this process drives sustained tissue-level propagation in human tumors remains limited.

Following internalization, aggregated p53 species can escape endosomal compartments and function as intracellular seeds that promote the conversion of soluble p53 into aggregation-prone conformations ^(10), (12)^. This intracellular seeding process enables pathological p53 assemblies to expand within recipient cells through nucleation-dependent recruitment of soluble p53 molecules. As aggregation progresses, β-sheet–rich fibrillar structures accumulate and form cytoplasmic inclusions that can persist as stable seeds for further aggregation. Such self-propagating assemblies may progressively disrupt intracellular proteostasis and interfere with p53-dependent signaling pathways. Consequently, the continued expansion of aggregation-prone p53 species within tumor cells may amplify oncogenic signaling networks and contribute to phenotypic heterogeneity within tumor cell populations. This propagation mechanism provides a potential explanation for how aggregation-prone p53 species expand within tumors and influence the behavior of surrounding cells.

## Clinical Evidence of the Amyloid State of p53 in Human Cancers

Although the aggregation of mutant p53 was initially characterized in biochemical and cellular systems, growing evidence indicates that amyloid-like p53 assemblies are present in human tumors and may have important clinical implications. Advances in pathological detection methods have enabled the identification of aggregated p53 species in patient-derived samples using approaches such as conformation-specific antibodies, amyloid-binding dyes, and biochemical fractionation techniques ^(30)^ (Table 2). One of the earliest demonstrations of p53 aggregation in clinical specimens came from studies showing the co-localization of mutant p53 with amyloid-like protein deposits in breast cancer tissues. Using amyloid-sensitive dyes and aggregation-specific antibodies, these studies revealed cytoplasmic inclusions containing aggregated p53 species. These observations provided the first direct evidence that aggregation-prone p53 structures identified in vitro can also occur in human malignancies.

**Table 2.** Clinical Evidence for Amyloid-Like or Aggregated p53 in Human Cancers.

Cancer type	Reference	Detection method	Sample size	Key finding/clinical association	Interpretation
Breast cancer	Levy et al., 2011	p53/amyloid/oligomer antibodies	Limited clinical specimens	Mutant p53 co-localized with amyloid-like protein deposits in breast tumor specimens.	Early proof-of-concept evidence for amyloid-like p53 in human cancer; no clinical outcome data.
Ovarian cancer	Heinzl et al., 2022	p53/amyloid/oligomer antibodies and p53-Seprion-ELISA	30 patients	Compared multiple technologies for detecting p53 aggregates in ovarian cancer tissue.	Methodological validation study but not designed to evaluate prognosis.
Ovarian cancer	Iwahashi et al., 2022	p53/ProteoStat co-localization	121 patients	Cytoplasmic p53 aggregates were associated with poor clinical prognosis and cisplatin resistance-related phenotypes.	Supports prognostic and functional relevance; retrospective single-institution cohort and selected aggregate-confirmation assays.
Ovarian cancer	Heinzl et al., 2023 (OVCAD consortium)	p53-Seprion-ELISA	81 patients	p53 aggregates were detected and were frequent in tumors with missense *TP53* mutations; clinical relevance was evaluated.	Independent external ovarian cancer cohort; assay standardization and mutation/context dependence remain important considerations.
Lung cancer	Nishitsuji et al., 2024	p53/ProteoStat co-localization	228 patients	Cytoplasmic p53 aggregates were associated with poor clinical prognosis and cisplatin resistance-related phenotypes.	Extends clinical relevance beyond ovarian cancer with larger cohort.
Nasopharyngeal cancer	Li et al., 2024	p53/amyloid/oligomer antibodies	90 patients	p53 amyloid aggregation was detected; p53-R280T promoted aggregation and impaired p53 nuclear function.	Recent cancer type-specific evidence; functional association is strong, but broader prognostic validation remains limited.
Glioma	Wu et al., 2025	Plasma detection of p53 aggregates by advanced single-molecule/aggregate approaches	190 patients	Demonstrated feasibility of detecting p53 aggregates in plasma of glioma patients.	Emerging non-tissue diagnostic approach; clinical utility and specificity require further validation.

ELISA: enzyme-linked immunosorbent assay.

Subsequent investigations have extended these findings to additional tumor types. In particular, high-grade serous ovarian carcinoma (HGSOC) represents a valuable model for studying p53 aggregation in clinical settings, as *TP53* mutations occur in the vast majority of cases. Pathological analyses have identified subsets of ovarian tumors that exhibit cytoplasmic p53 aggregates, detectable through aggregation-sensitive staining approaches and biochemical analyses ^(31)^. Importantly, the presence of aggregated p53 appears to correlate with clinical outcomes. Analyses of ovarian cancer cohorts have demonstrated that tumors harboring cytoplasmic p53 aggregates are associated with significantly poorer progression-free survival and overall survival compared with tumors lacking detectable aggregates ^(32), (33)^. These findings suggest that the conformational state of p53, rather than simply the presence of *TP53* mutations, may influence tumor behavior. Evidence of p53 aggregation has also been reported in other malignancies, including lung cancer, breast cancer, colorectal cancer, salivary duct cancer, and oral squamous cell cancer ^(34), (35), (36), (37), (38)^. More recently, amyloid aggregation induced by the p53-R280T mutation was reported in nasopharyngeal carcinoma, where aggregated p53 was associated with impaired nuclear localization and loss of p53 transcriptional activity ^(39)^. In these contexts, aggregation-prone p53 variants may promote aggressive tumor phenotypes by disrupting transcriptional regulation and altering tumor suppressor networks. The ability of mutant p53 aggregates to sequester structurally related proteins such as p63 and p73 may further amplify these oncogenic effects.

Another clinically relevant consequence of p53 aggregation may involve therapeutic resistance. Aggregated p53 assemblies can interfere with apoptotic signaling pathways and stress-response networks that normally contribute to treatment-induced cell death ^(12), (32), (34)^. Experimental studies have suggested that tumor cells harboring aggregation-prone p53 variants may exhibit reduced sensitivity to chemotherapy and radiation therapy, although the precise relationship between p53 aggregation and therapeutic response remains an active area of investigation. From a diagnostic perspective, the detection of p53 aggregates may provide new opportunities for biomarker development. Conventional p53 immunohistochemistry is widely used in pathology to infer *TP53* mutation status based on patterns of protein accumulation or absence ^(40), (41)^. However, this approach does not capture the conformational state of the protein. Incorporating methods capable of identifying amyloid-like p53 assemblies may therefore improve the stratification of *TP53*-mutant tumors and help identify patient subsets with distinct biological behaviors.

Taken together, these clinical observations support the view that p53 aggregation is a biologically and clinically relevant phenomenon in human cancer. The presence of aggregated p53 in tumor tissues, its association with aggressive disease features, and its potential influence on therapeutic responses highlight the importance of considering the structural state of p53 when evaluating *TP53*-mutant malignancies.

## Therapeutic Targeting of the Amyloid State of p53

The recognition that selected mutant p53 proteins can adopt aggregation-prone amyloid-like states has opened new possibilities for therapeutic intervention. Historically, mutant p53 has been considered a difficult drug target because many *TP53* mutations impair DNA binding without generating enzymatic active sites that can be readily inhibited by small molecules ^(42), (43), (44)^. The emerging view of mutant p53 as a protein misfolding–driven oncogenic state suggests alternative strategies focused on protein conformation, aggregation dynamics, and proteostasis. Therapeutic approaches should be distinguished according to whether they directly target p53 aggregation or more broadly modulate mutant p53 stability and function ^(10), (17), (45)^. Direct anti-aggregation strategies, such as ReACp53 and related peptide-based inhibitors, are designed to interfere with aggregation-prone segments and suppress amyloid-like assembly. ReACp53, a cell-penetrating peptide designed to inhibit mutant p53 amyloid formation, has been shown to restore p53 function in cancer cell lines and patient-derived organoids, including HGSOC models ^(46)^. Experimental studies have shown that inhibition of p53 aggregation can induce apoptosis in tumor cells and suppress tumor growth in preclinical models ^(32), (34), (46), (47), (48)^. In contrast, conformational rescue agents such as PRIMA-1 and APR-246/eprenetapopt aim to restore mutant p53 function or modulate cellular redox balance and should not be regarded as aggregation-specific therapies ^(34), (49), (50), (51)^. APR-246/eprenetapopt showed promising activity in early-phase studies in *TP53*-mutant myelodysplastic syndromes and acute myeloid leukemia when combined with azacitidine ^(52), (53)^. More recent follow-up data have further informed its clinical development in *TP53*-mutant myeloid malignancies ^(54)^. However, subsequent clinical development has underscored the challenges of translating mutant p53 reactivation strategies into consistent clinical benefit. Therefore, APR-246 should be discussed as a broader mutant p53–reactivating and redox-modulating agent rather than as a specific anti-aggregation therapy, and its clinical development should be interpreted cautiously.

Targeting the cellular proteostasis network provides an additional strategy. Many tumor cells rely on molecular chaperones, particularly HSP90, to stabilize mutant p53 proteins ^(55), (56), (57)^. Inhibition of the HSP90 chaperone system can destabilize mutant p53 and promote its degradation through the ubiquitin–proteasome pathway. Such approaches exploit the dependency of tumor cells on stabilized mutant p53 for survival. Another potential strategy involves interfering with the intercellular propagation of p53 aggregates. As discussed in previous sections, HSPGs can facilitate the uptake of extracellular p53 aggregates ^(12), (26)^. Disrupting interactions between aggregated proteins and glycosaminoglycans may therefore limit the spread of aggregation-prone p53 conformations within tumor tissues. Finally, therapeutic strategies that enhance protein quality control systems, including autophagy and proteasomal degradation pathways, may help eliminate aggregated p53 species ^(58), (59), (60)^. Pharmacological modulation of these pathways could reduce the accumulation of pathogenic aggregates and restore cellular proteostasis.

Together, these approaches highlight multiple opportunities to therapeutically target the amyloid state of mutant p53. By inhibiting aggregation, restoring native folding, destabilizing mutant proteins, or blocking aggregate propagation, it may be possible to transform one of the most common oncogenic drivers in human cancer into a tractable therapeutic target.

## Conclusion and Future Perspectives

The concept that mutant p53 can adopt an amyloid-like aggregated state provides a new framework for understanding the biology of *TP53*-mutant cancers. Traditionally, mutant p53 has been viewed primarily through the lens of genetic alteration, with tumor progression attributed to loss of tumor-suppressive activity and gain-of-function signaling pathways. The emerging evidence discussed in this review suggests that an additional layer of regulation exists at the level of protein conformation and aggregation state. Structural studies have revealed that many cancer-associated mutations destabilize the p53 DNA-binding domain and expose aggregation-prone sequences that drive amyloid formation. These aggregates can recruit additional p53 molecules through templated misfolding mechanisms and may propagate within tumor tissues through interactions with the extracellular microenvironment. In this context, mutant p53 shares several mechanistic features with proteins involved in neurodegenerative proteinopathies, including the formation of β-sheet–rich aggregates and the potential for prion-like propagation. Recognition of mutant p53 as a conformational proteinopathy has important implications for cancer biology. Aggregation of mutant p53 may influence multiple aspects of tumor behavior, including transcriptional regulation, cellular stress responses, and interactions with the tumor microenvironment. The ability of aggregated p53 to sequester members of the p53 protein family, such as p63 and p73, further amplifies its oncogenic effects.

Despite these advances, several key questions remain unresolved. The precise structural organization of p53 aggregates in human tumors remains incompletely characterized, and the factors that determine whether mutant p53 adopts soluble, oligomeric, or fibrillar states are not fully understood. In addition, the extent to which intercellular propagation of p53 aggregates contributes to tumor heterogeneity and disease progression requires further investigation. Future studies integrating structural biology, cancer genomics, and spatial pathology will be essential for addressing these questions. Advances in imaging technologies and proteomic approaches may enable direct visualization and characterization of p53 aggregates in clinical specimens ^(61), (62), (63)^. Such approaches could help determine how the conformational state of p53 influences tumor evolution and therapeutic response. From a translational perspective, targeting the aggregation state of mutant p53 represents a promising therapeutic strategy. Approaches aimed at inhibiting aggregation, restoring native protein folding, destabilizing mutant p53, or blocking intercellular propagation may provide new avenues for the treatment of *TP53*-mutant cancers.

Taken together, the emerging view of mutant p53 as an aggregation-prone oncogenic protein highlights a previously underappreciated dimension of cancer biology. Understanding how protein misfolding and conformational propagation contribute to tumor progression may ultimately open new opportunities for diagnosis, prognosis, and therapy in cancers driven by *TP53* mutations.

## Article Information

### Author Contributions

Conceptualization: Naoyuki Iwahashi and Kazuhiko Ino; writing―original draft: Naoyuki Iwahashi; writing―review and editing: Kazuhiko Ino; visualization: Naoyuki Iwahashi; supervision: Kazuhiko Ino; project administration: Naoyuki Iwahashi.

### Conflicts of Interest

None

### Article Information

This article is based on the study that received the Medical Research Encouragement Prize of the Japan Medical Association in 2025.
